# Evaluation of the Effectiveness of Therapy for Anxiety in Williams Beuren Syndrome Using a Smartphone App: Protocol for a Single-Case Experiment

**DOI:** 10.2196/44393

**Published:** 2023-04-03

**Authors:** Natacha Lehman, Raphaël Trouillet, David Genevieve

**Affiliations:** 1 EA4556 Laboratoire Epsylon Université Paul Valery Montpellier 3 Montpellier France; 2 Genetic Department Montpellier Hospital Montpellier France; 3 Institut National de la Santé et de la Recherche Médicale U1183 Montpellier University Montpellier France

**Keywords:** single case experimental design, cognitive behavioral therapy, intellectual disability, rare disease, Williams syndrome, smartphone application, ecological momentary assessment

## Abstract

**Background:**

Williams syndrome (WS-OMIM 194050, orphaned number: Orpha 904) is a rare condition mostly associated with intellectual disability. People with Williams syndrome are 8 times more likely to have anxiety disorders than the general population. Therapeutic solutions to treat the anxiety remain limited, particularly nonpharmacological therapy. However, cognitive behavioral therapy (CBT) has been found efficacious in managing anxiety disorders and can be used for people with intellectual disability.

**Objective:**

This paper describes a protocol to assess the efficiency of a CBT program based on digital support for people with Williams syndrome and anxiety based on a research methodology designed for rare diseases.

**Methods:**

We will recruit 5 individuals with Williams syndrome and anxiety. They will participate in 9 CBT sessions. Participants will perform daily self-assessments of anxiety using a digital app, which will allow for ecological and repeated evaluation of their anxiety. This digital app will provide support for each therapy session. Anxiety and quality of life will be externally assessed before and after the program and at a 3-month follow-up. This is a single-case intervention research design with multiple baselines implying repeated measures of judgment criteria. The present protocol ensures high internal validity and will help identify encouraging contributions for later clinical trials.

**Results:**

Participant recruitment and data collection began in September 2019, and we project that the study findings will be available for dissemination by spring 2023.

**Conclusions:**

This study will allow the assessment of the efficiency of a CBT program based on digital support to treat anxiety in people with Williams syndrome. Finally, the program could be used as an example of nonpharmacological therapy for rare diseases.

**Trial Registration:**

ClinicalTrials.gov ID: NCT03827525; https://clinicaltrials.gov/ct2/show/NCT03827525

**International Registered Report Identifier (IRRID):**

DERR1-10.2196/44393

## Introduction

Williams syndrome (WS) is a rare neurodevelopmental disorder caused by a 7q11.23 deletion encompassing about 25 genes on chromosome 7 [1]. The estimated prevalence is 1 in 7500 live births [2]. Most individuals present with intellectual disability and peculiarities in neuropsychological functioning. The cognitive profile is heterogeneous in terms of strengths and weaknesses and in global functioning [3]. Language skills are generally good, often with deficits in certain executive functions (especially inhibition) [4].

The prevalence of anxiety disorders in WS is high, varying from 16% to 82% [5]. With an anxiety rate 8 times higher than in the general population and 4 times higher than in other individuals with intellectual disability [6], it is the most prevalent mental health problem for individuals with WS. The available drug treatments have been found effective in reducing the level of anxiety in individuals with WS. However, they have side effects [7]. Despite the consequences on quality of life (QoL), there is a lack of research on therapeutic protocols for anxiety for people with WS.

Cognitive behavioral therapy (CBT) belongs to a class of scientifically validated therapeutic interventions. It aims to modify dysfunctional ways of thinking, as well as behavioral patterns, to reduce psychological problems. CBT interventions for anxiety share a focus of changing dysfunctional beliefs and anxious anticipation by using different cognitive, behavioral, and emotional techniques [8]. A meta-analysis [9] showed that CBT provided greater clinical benefit than other forms of psychotherapy in treating several psychological disorders in individuals with intellectual disability. However, in the context of intellectual disability, the number of participants recruited in these studies remains low, and the methodologies used have numerous limitations (eg, no controlled manipulation of the treatment delivered, studies with only 1 patient [10-12], studies evaluating the impact of therapy during 1 week of hospitalization, studies do not allow for patient empowerment [13]). Many of the results tend to show that CBT is a promising therapy for treating the anxiety of patients with intellectual disability [14], but the few studies available and their methodological limitations make further research necessary [15].

A pilot study carried out in 2013 highlighted a mindfulness-based intervention for individuals with WS [13]. The practice of mindfulness is frequently used in CBT interventions. It teaches one to pay attention and to have moment-to-moment awareness [16]. This CBT treatment approach has been found efficacious in treating emotional disorders in individuals with WS. This pilot research took place in a 5-day institutional setting, and the long-term effects of the treatment were not evaluated. In addition, the application of treatment in a context of institutionalization raises the question of the generalization of the results and the autonomy of individuals with WS.

Another study was conducted in 2022, in which a group of 4 adults with WS were virtually delivered an adapted CBT intervention [17]. In this study, a CBT program was adapted for people with intellectual disability. Participants and caregivers in this study reported a high level of satisfaction and a reduction in their anxiety. This study provided support for the acceptability and feasibility of group-based CBT treatment for anxiety in WS. However, the sample size was small, and the methodology used did not have strong scientific reliability.

The objective of our study is to propose and discuss clinical interest in CBT with digital support for anxiety in people with WS, taking into account intellectual disability. Because of the rarity of WS, the single-case methodology seems appropriate. In addition, this methodology allows for analyzing the effects of the intervention at an individual level. The development of digital support will allow participants to perform the therapy exercises at home. This could be particularly interesting for people with intellectual disabilities who need to be assisted with the realization of a number of daily tasks. The objective of the digital support is to empower them. Furthermore, this application makes it possible for the patient to evaluate himself or herself from home on a daily basis, allowing an ecological repeated assessment. We hypothesize that patients with WS will be able to engage in an adapted CBT protocol. We hypothesize that individuals’ anxiety will decrease over the course of therapy. We also hypothesize that other factors such as QoL will be improved with this therapy protocol.

## Methods

### Study Design

We will use a multiple baseline AB single-case experimental design across participants to evaluate the efficacy of the intervention. This method allows for manipulating the intervention in an experimentally controlled manner and testing causal relations among variables of interest in a small group of participants. The multiple baseline design is well-suited for interventions that are irreversible due to learning effects, and it enables testing of whether the improvement in mental health is due to therapy or random factors changing with time [18]. For this purpose, we will use a multiple baseline across subjects and across behaviors design, as our patients will complete a daily self-assessment of 6 items related to their emotions. We will recruit only 5 patients because WS is a rare disease and a minimum of 3 replications of the effect is recommended to increase the reliability of our findings [19]. For each participant, we will first obtain repeated measures during the baseline phase (A) when no treatment is provided. Patients will be assigned to different lengths of the baseline phase to ensure at least 3 replications of the treatment effect [20]. The possible duration of the baseline phase ranges from 14 days to 30 days before treatment onset, as a minimum of 5 measurement points is required [21]. These repeated measures during the baseline phase serve as control conditions to evaluate what changes can be expected without the treatment [9,22]. The duration of the baseline will be randomly assigned by the experimenter. Following that, repeated measures are obtained during a 9-week treatment phase (B) and during 3 months after the treatment to ensure that our measures did not return to the baseline level after the treatment has been withdrawn. Patients will also complete a range of nomothetic outcome measures 3 times: (1) baseline, (2) intervention, and (3) follow-up termination.

### Participants

We will recruit 5 participants for this study. The inclusion criteria are age >18 years, molecular confirmation of WS, sufficient level of language assessed by a score >7 on the Clinical Evaluation of Language Fundamentals 4 test [23], and complaints of anxiety. The complaints of anxiety can be expressed by the individual or the individual’s representative. For the inclusion criteria in this study, we chose to evaluate this complaint with the following Likert scale completed by the participant: “This week I have been anxious” or “This week the individual has been anxious.” To be included in the protocol, the participant will need to score at least 1 on this self-rated Likert scale. We chose to include people with low anxiety as well as high anxiety, even if an anxiety disorder is not characterized, because anxiety is generally underestimated in WS, with one longitudinal study finding co-occurring anxiety in approximately 82% of patients with WS [24].

Exclusion criteria are uncorrected visual disturbance, uncorrected deafness, or being already treated for anxiety. Because of the intellectual disability, individuals with WS will be accompanied by a caregiver during visits requiring signing consent or a hetero-evaluation.

Individuals will be recruited from patient associations and among individuals followed in the medical genetics department of Montpellier University Hospital. Interested individuals will receive information about the study, first with a letter; then, they will be in contact with a doctor involved in the study to ask questions. Because of the rarity of WS, individuals recruited could live far from the hospital where the study is taking place. Financial support for travel costs will be offered to each individual taking part in this study. Individuals with WS who wish to be involved and a legal representative will sign written informed consent; then, the individual will be prescreened for inclusion and exclusion criteria.

### Ethical Approval

The study has been registered with the committee of protection of people (CPP) Sud-Ouest et Outre-Mer 1 (approval number CPP 1-18-86/SI:1752 (2° HPS)).

### Procedures

#### Baseline

We will recruit 5 individuals with WS. Each participant will have a different length of the baseline period (A). The participants will be allocated to a baseline lasting from 2 weeks to 5 weeks. The baseline phase will begin at the same time for each participant. This multiple baseline design allows for the multiple assessments to serve as control conditions to evaluate what changes can be expected without the treatment [22]. During this baseline period, no therapeutic intervention will take place. The participants will continue their daily life without changing their habits. They will complete a daily self-assessment of 6 items related to their emotions.

#### Intervention

After the baseline period, each individual will undergo a psychotherapeutic intervention with digital support. This intervention will extend over 9 sessions, spaced 1 week to 2 weeks apart depending on the individual’s availability. Each session will last from 45 minutes to 90 minutes and will be carried out with the same clinical psychologist. Participants will use a smartphone app daily as support for the therapy. The app will include relaxation exercises and mindfulness audio. Individuals will continue their daily self-assessment during this intervention period.

#### Follow-up

After the 9 intervention sessions, individuals will be invited to continue to apply tools delivered during the intervention in their daily life. They will continue their daily self-assessment for 3 months after the intervention.

During the research (baseline, intervention, follow-up), the individual’s representative will complete daily evaluations.

**Figure 1:**
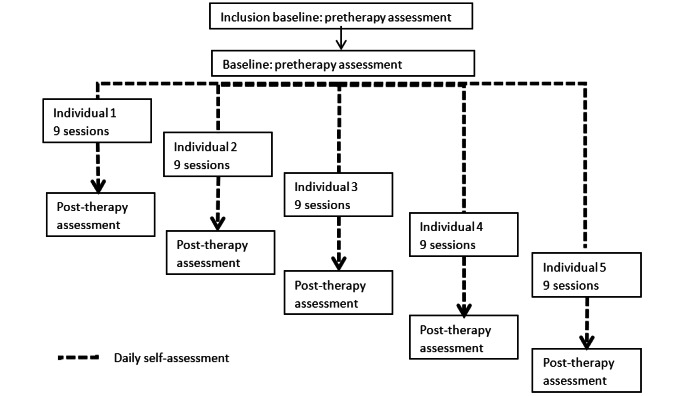
Protocol flow.

Hetero-evaluations will be carried out during the inclusion visit (baseline), the visit at the start of the intervention (T1), the visit at the end of the intervention (T9), and the follow-up visit 3 months after the end of intervention (Figure 1).

### Measures

#### Repeated Measures: Real-Time Monitoring Technology

**Figure 2:**
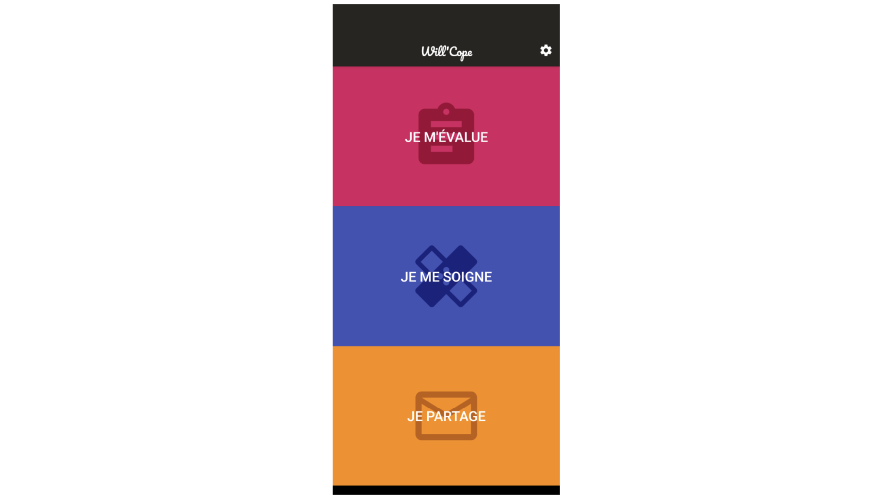
Screenshot of the WillCope app.

**Figure 3:**
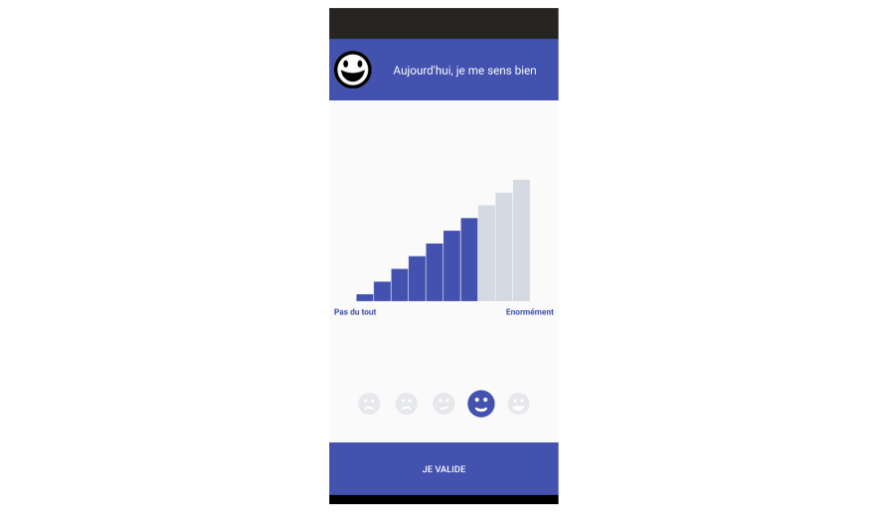
Screenshot of the WillCope app.

There is no validated tool to assess anxiety repeatedly in our target population, to our knowledge [25]. To take into account the specificity of a population with intellectual disability, a smartphone tool has been developed to allow for repeated self-assessment. It is an app named WillCope. Screenshots of this app are presented in Figures 2 and 3. This app includes 6 questions, each related to anxiety manifestations that are usually used to assess anxiety. The items evaluated were initially chosen by a working committee of 10 people including doctors, psychologists, patients, and caregivers. The choice of these items was based on the Hamilton Rating Scale for Anxiety (HAM-A) [26-28]. Items and questions were tested in a study of >300 persons with intellectual disability.

We will use a single item to measure each targeted behavior because it can be more easily included in an experiment in which patients have to perform daily self-assessments for several months. In addition, single items have been shown to be as reliable as assessments using longer scales [29]. In this study, 6 questions will be presented to the 5 individuals, who will evaluate the intensity of each of the items on a daily basis using a Likert scale from 1 to 10. These 6 questions make it possible to evaluate the principal components encountered in anxiety disorders in people with intellectual disability, taking into account the somatic dimension. The wording of the questions is in easy-to-understand language and is associated with a pictogram as well as an audio reading. The questions are formulated in French and are related to anxiety, pain, sleep, motor agitation, well-being, and motivation.

Regarding anxiety, Williams et al [30] demonstrated the scientific validity of a single-item scale for assessing anxiety: “Today, I am anxious.”

Pain is included because of the importance of somatic symptoms associated with anxiety: “Today, I have pain in my body.”

Sleep is included because of the impact of anxiety on sleep: “Today, I slept well.”

Motor agitation is part of the manifestations of anxiety, especially with intellectual disability: “Today, I need to move around.”

Well-being is directly affected by anxiety: “Today, I am happy.”

Motivation can also be affected by anxiety: “Today, I feel like doing things.”

The primary outcome will be the score obtained for the first question related to anxiety.

The neuropsychologist will check that the questions are correctly understood during the first evaluation. A support person may also accompany the individual in the daily evaluation for guidance, without answering for the individual.

A repeated assessment questionnaire will also be provided to the support person or the caregiver accompanying the individual, to obtain a repeated hetero-assessment of the individual's anxiety. The questions will be the same as in the self-evaluation but will be formulated in the third person.

The smartphone support allows for real-time monitoring of the emotional state of individuals in an ecological environment in their daily life. Patients with intellectual disability might have difficulties self-assessing using classical CBT tools like charts to complete or notebook-based assessments completed with paper and pencil. The app was developed in easy-to-read and understand language, with pictograms and audio support, which makes it accessible to people who cannot read or write. An alarm is integrated into the app to remind the participants to complete the exercises. A backup of each of the patient’s self-assessment is encrypted and stored in the app. It can then be shared by email but requires decryption software to be opened. This backup allows visibility on the frequency of use of the app for self-assessment.

#### Nomothetic Quantitative Measures

Nomothetic quantitative measures will be completed at the assessment, termination, and follow-up visits.

A hetero-assessment of anxiety will be performed by a clinical psychologist using the HAM-A [26,27]. This scale is the most widely used test to assess the intensity of anxiety symptoms. It is applicable to all adults and consists of 14 questions rated according to the severity of symptoms: anxious mood (worry, apprehension, irritability); feelings of tension, fatigability, inability to relax, startled reactions, crying, tremors, fears; sleeping troubles; difficulty concentrating and memory problems; depressed mood; somatic symptoms; and behavior during the interview. Symptoms are rated using 5 degrees of severity, from absence to disabling intensity. The overall score ranges from 0 to 56: the higher the score, the more severe the anxiety. This scale will be administered by a clinical psychologist.

This scale does not constitute our main criterion of analysis and will be used in addition to the single-item repeated measures of anxiety-related behaviors and manifestations. It seems to us interesting to allow a descriptive analysis of the evolution of anxiety before and after treatment, and this scale presents several advantages for evaluating anxiety in a population with intellectual disability. Indeed, it allows a hetero-evaluation of anxiety by a psychologist. It also allows the somatic dimension of anxiety, as well as the patient's behavior with its anxious manifestations, to be taken into account. QoL will be assessed with the Abbreviated World Health Organization Quality of Life (‎WHOQOL-BREF) questionnaire [31]. This scale consists of 26 items, each rated from 0 to 5. The scale will be completed by the representative and by the individual with WS assisted by a clinical psychologist who can explain the items that would be difficult to understand.

#### Ideographic Qualitative Measures

Individuals will undergo a semidirected interview with a psychologist at each session to identify any significant events in the patient’s life. This interview can last from 5 minutes to 30 minutes. The objective will be to observe the presence of factors that cannot be measured by quantitative analyses. The main themes addressed will be the following: the occurrence of a significant event in the participant’s life, daily activities, relationships and social situation, autonomy. We will also observe the perception of the impact of the therapy on daily life and elements of daily life modified thanks to the therapy.

In addition, a questionnaire will be completed by the patient at the end of the research and at 3 months after the therapy to evaluate the benefit of and satisfaction with the therapy. The semistructured interviews will be transcribed verbatim. We will determine the presence or absence of the aforementioned factors. The data will be extracted and combined with the data from the questionnaire completed at the end of the therapy and at the follow-up, to evaluate the effectiveness of the intervention.

### Intervention

In our study, the intervention will consist of a CBT program based on a process-based approach targeting the processes underlying emotional disorders [32]. This protocol has the advantage of targeting cognitive processes common to different forms of anxiety disorders. Anxiety disorders can have different manifestations in WS, and the rarity of this disease does not allow us to recruit a sufficient number of patients with the same clinical picture. Thus, this protocol is particularly suitable to target the cognitive anxiety processes common to all our patients despite their differences in expression. We will build on the unified protocol designed for children and first developed in 2011 [33]. This protocol has been adapted for WS based on existent literature [30]. It has been found that people with intellectual disability can perform better when linking situations directly to emotions than they do when attempting to link the triad of beliefs, emotions, and situation [34]. It has been shown that the identification of behavior and feelings is associated with verbal ability and the identification of thoughts is linked to IQ [35]. Language ability is very developed in people with WS, but their ability to understand some abstract situations is more limited. It has been shown that the behavioral elements are more consistently effective than the cognitive elements and the behavioral elements are more amenable to short-term intervention [36], which is the case in our study. For these reasons, we decided to not emphasize cognitive restructuration in our protocol but to focus on emotion identification, exposure, and meaningful actions.

During the entire protocol, the psychologist will speak slowly and repeat information. He will give the participant time. He will only emphasize the essential points and give concrete examples. The content of each module is simplified and adapted in easy-to-read and understand language in a way that empowers individuals with WS. Nonverbal communication is emphasized.

People with intellectual disability often have poor self-image, so the following points, which are important in any psychotherapy work, are particularly so with this population. The therapeutic alliance is very important. The patient must be involved in the choice of exercises to be done at home. The objectives must be simple and achievable. Throughout the work, positive reinforcement and appreciation of the participant are essential.

A clinical psychologist specializing in CBT will perform the intervention during the 9-session therapy protocol. Each session will last about 1.5 hours and will be organized as described in Table 1.

First, mindfulness, which has been found effective in individuals with WS, will be integrated into each session [37]. Mindfulness is our innate capacity to pay conscious and full attention to something in the moment. It is often defined as “the awareness that emerges through paying attention on purpose, in the present moment, and nonjudgmentally to the unfolding of experience” [16]. Usually, anxiety therapy is based on mindfulness-based stress reduction. In this protocol, we adapted mindfulness exercises with some exercises used with children [38-40]. The main exercises are mindfulness of the body, of the breath, and of the senses. Mindfulness exercises are integrated into the app so that the participant can repeat them from home on a daily basis. The protocol will target increasing awareness of the emotions present as well as tolerance to emotional sensations. It will also aim to increase cognitive flexibility and will identify and prevent emotional avoidance behaviors as well as exposure.

The intervention will require the daily use of the WillCope app. In addition to allowing daily assessment by the patient, the WillCope app will provide therapy support through relaxation and mindfulness tools. The presence of a single support to allow self-assessments, as well as the realization of mindfulness and relaxation exercises, is facilitating for patients.

**Table 1:** Main stages of therapy.

Stage of therapy				Description
Retrospective				Each session begins with a look back at the past week and a return to the exercises performed at home.
**Mindfulness and relaxation**				
	Overall		In every session, individuals with WS^a^ will be taught how to perceive their body, thoughts, and emotions objectively, in the present moment, without judging them. Mindfulness helps to stay with difficult feelings without analyzing, suppressing, or encouraging them. Individuals with WS often report that their emotions are out of their control, suddenly invading them in a confusing, disturbing way. This module will help individuals recognize the interaction between thoughts, feelings, and behaviors during the emotional experience. The therapist will help identify primary reactions, or early emotional responses to a situation or memory, and reactions to these primary emotions, which tend to be negative and not focused on the present moment. These concepts are taught during the sessions using concrete emotional situations, activating experiences adapted to each individual. Specifically, reactions to emotions tend to be subjective, judgmental, and negative. An example is a concern that anxiety could hinder the smooth running of an activity in the future. To the extent that these reactions are not based on actual information in the present moment, they may block positive information about the nature of emotional responses.	
	**Unit 1: functional analysis (3 sessions)**			
		Problem identification	What are the triggers, in what context, what are the associated behaviors and thoughts; intensity of emotions; duration of emotions; consequences of associated behaviors	
		Identification of motivational elements	What are the individual's values, what are the things that make him happy, and what does he want to develop in his life?	
		Realization	A schematic illustrating the preceding points is reviewed with the individual.	
	Unit 2: psychoeducation (3 sessions)		This module consists of a psychoeducation session on emotions in general and anxiety in particular. Emotions such as anger, sadness, and fear are described. During this module, the therapist explains the cognitive, physiological, and behavioral components of emotions and their interactions. Individuals should then begin to understand that their emotions have an adaptive and functional role, providing information about the environment and motivating certain actions. The therapist can describe how avoidance during the emotional event (consequence) leads to maintenance of the anxiety and distress associated with the emotional experience. Schematics are built to illustrate the components of the different emotions of the individual, based on experience in daily life	
	Unit 3: exposure and engagement in meaningful actions (3 sessions)		Previous sessions allowed the individual to become familiar with feelings related to emotions. One of the difficulties with anxiety is a limitation of the individual's activities and autonomy due to avoidance. Therefore, the last sessions of the therapy are devoted to setting up exposure and actions that allow the individual to maintain autonomy and engage in actions that count, even if unpleasant emotions are present. In each session, examples are applied to the individual's daily life. Tools are provided to deal with some difficult emotions. For example, squeezing a frozen lemon in the hands can defocus the attention of anxiety or anger and defer an impulsive response.	
Representative				At the end of each session, the tools presented during the session are described to the individual’s representative.
At home				Exercises to be carried out daily in connection with the current module. Every day, the individual with WS must continue to perform self-evaluation as well as a relaxation or mindfulness exercise available on the WillCope app.

^a^WS: Williams syndrome.

### Data Analysis

Our primary outcome will be the changes in the level of anxiety evaluated with a repeated scale. We will graph and analyze data for each participant and each target behavior. We will first visually inspect the analyses as per the guidelines for visual analysis in single-case studies [23]. Within-condition analysis (baseline and intervention phases) will include the evaluation of the stability of the level of data by condition by calculating means, medians, ranges, and stability envelopes. The absolute level of change will be evaluated by calculating the difference between the first and last values in each condition. Between-condition analysis of data will allow for comparisons of adjacent data in the study. The change in the trend direction between phases A and B will be evaluated by identifying the trend as accelerating, decelerating, or zero-celerating. The therapeutic or contra-therapeutic direction will also be reported. The level of change between conditions will be evaluated by calculating the median level of change.

A comparative table of data will be edited for each repeated measure, patient, and step of the protocol. The means, medians, range, stability envelope, and absolute level of change will be presented in this table.

We will use the Tau-*U* to examine the within-phase trend and between-phase difference to estimate the treatment effect both within and between phases. We prefer the Tau-*U* to alternative statistics (ie, proportion of nonoverlapping data) because it provides a baseline trend–corrected estimation of trends in treatment and follow-up phases by combining nonoverlap between phases and trends within phases. The following 3 Tau-*U* indices will be calculated: (1) Tau-*U* A-to-A compares a phase-A score with another phase-A score, (2) Tau-*U* B-to-B compares a phase-B score with another phase-B score, and (3) Tau-*U* A-to-B compares a phase-A score with a phase-B score. The Tau-*U* A-to-B is similar to the Mann-Whitney *U* test for group independence, and a *P* value is produced in addition to an estimate of effect size. These Tau-*U* indices estimate the effect of the treatment on both within phase trends and between-phase differences. We will use the following 2 forms of the Tau-*U* indices: the uncorrected and corrected Tau-*U*. The corrected Tau-*U* estimates the degree of the baseline trend and adjusts baseline and treatment phase observations to remove the effect of the baseline trend. We will use the corrected Tau-*U* to estimate an effect size from the corrected data when the Tau-*U* A-to-A is significant [41]. We will use the Tau-*U* calculator web-based application [42].

We will estimate the reliability of change for our nomothetic outcomes using the reliable change index (RCI) [43]. The RCI measures in which direction an individual has changed and estimates whether this change is reliable and clinically significant. We will calculate the RCI statistic for the 3 dimensions of the French WHOQOL-BREF (physical health, psychological health, and social relationships) for which reliability coefficients are available [31]. For each of these 3 WHOQOL-BREF dimensions, we will calculate the value of the standard using our patients’ scores at baseline. A reliable change in both treatment and follow-up compared with baseline measures will be achieved with values ≥1.96.

Concerning the HAM-A, there is no fidelity index validated in French, which does not allow the calculation of an RCI value. The results obtained from this evaluation will therefore be used in a descriptive manner, in order to relate them to the repeated evaluations of anxiety that constitute our main evaluation criterion.

A comparative table data will also be edited with the nomothetic outcome measures.

## Results

As of spring 2019, the app was fully developed. Participant recruitment and data collection began in September 2019. Due to the COVID-19 crisis, data processing was postponed in 2022-2023, and it is projected that the study findings will be available for dissemination by summer 2023.

## Discussion

In this manuscript, we describe the research protocol for evaluating an anxiety therapy program for individuals with WS. WS is a rare genetic disease in which individuals present with intellectual disability of variable intensity but, in general, have good language skills. Most individuals with WS present with anxiety. Because of the rarity of WS, there is a lack of validated studies on the psychotherapy of anxiety in these individuals. Therefore, their anxiety is usually treated with medication, and the side effects are more severe than in the general population. However, some therapies have been found effective in individuals with intellectual disability. Because of their good language skills, such individuals could particularly benefit from psychotherapy.

This manuscript describes the research protocol to test the effectiveness of CBT for anxiety in individuals with WS. This protocol is based on the single-case and multiple baseline research methodology. This methodology is particularly interesting for 2 aspects. First, it is a scientific research method that applies to small samples (as few as 3 people), so it is a particularly interesting methodology for rare diseases. Second, this methodology enables consideration of interindividual variations by observing the results of each individual individually. It enables evaluation of the fluctuations of anxiety in the daily life of individuals. For this protocol, a smartphone app was developed. This app allows daily self-assessment of anxiety by the patients and can be a support mechanism for the therapy protocol. This app can be particularly helpful for a population with an intellectual disability, for whom the appropriation of certain CBT tools may be difficult.

However, this work has some limitations. First, regarding the app, some improvements might be possible. The developed app has not yet been validated from a psychometric point of view. It can only be used to assess the evolution of anxiety for each patient but cannot be used as a diagnostic tool.

In addition, this app makes it possible to store the participant's evaluation data and thus to know when the patient evaluated his or her anxiety, but there are no data to know how the participant used the other tools in the app (the therapy support tools). It would be interesting to integrate a module to know how often the participant uses the app for therapeutic purposes.

Finally, this protocol only includes a small sample of patients with WS. Depending on the results, it would be interesting to evaluate this protocol with patients with other pathologies.

## Abbreviations

CBT: cognitive behavioral therapy
CPP: committee of protection of people
HAM-A: Hamilton Anxiety Rating Scale
QoL: quality of life
RCI: reliable change index
WHOQOL-BREF: Abbreviated World Health Organization Quality of Life
WS: Williams syndrome
